# Mobile Technology–Based Interventions for Stroke Self-Management Support: Scoping Review

**DOI:** 10.2196/46558

**Published:** 2023-12-06

**Authors:** Alexandra N Thompson, Deirdre R Dawson, Jean Michelle Legasto-Mulvale, Nivetha Chandran, Chelsea Tanchip, Veronika Niemczyk, Jillian Rashkovan, Saisa Jeyakumar, Rosalie H Wang, Jill I Cameron, Emily Nalder

**Affiliations:** 1 Rehabilitation Sciences Institute Temerty Faculty of Medicine University of Toronto Toronto, ON Canada; 2 Rotman Research Institute Baycrest Health Sciences Toronto, ON Canada; 3 Department of Occupational Science and Occupational Therapy Temerty Faculty of Medicine University of Toronto Toronto, ON Canada; 4 Department of Physical Therapy Temerty Faculty of Medicine University of Toronto Toronto, ON Canada; 5 School of Rehabilitation Science Faculty of Health Sciences McMaster University Hamilton, ON Canada; 6 KITE Research Institute Toronto Rehabilitation Institute University Health Network Toronto, ON Canada

**Keywords:** stroke, chronic disease, self-management, rehabilitation, mobile health, mHealth, eHealth, telehealth, telemedicine, digital health, mobile phone

## Abstract

**Background:**

There is growing interest in enhancing stroke self-management support using mobile health (mHealth) technology (eg, smartphones and apps). Despite this growing interest, “self-management support” is inconsistently defined and applied in the poststroke mHealth intervention literature, which limits efforts to synthesize and compare evidence. To address this gap in conceptual clarity, a scoping review was conducted.

**Objective:**

The objectives were to (1) identify and describe the types of poststroke mHealth interventions evaluated using a randomized controlled trial design, (2) determine whether (and how) such interventions align with well-accepted conceptualizations of self-management support (the theory by Lorig and Holman and the Practical Reviews in Self-Management Support [PRISMS] taxonomy by Pearce and colleagues), and (3) identify the mHealth functions that facilitate self-management.

**Methods:**

A scoping review was conducted according to the methodology by Arksey and O’Malley and Levac et al. In total, 7 databases were searched. Article screening and data extraction were performed by 2 reviewers. The data were analyzed using descriptive statistics and content analysis.

**Results:**

A total of 29 studies (26 interventions) were included. The interventions addressed 7 focal areas (physical exercise, risk factor management, linguistic exercise, activities of daily living training, medication adherence, stroke education, and weight management), 5 types of mobile devices (mobile phones or smartphones, tablets, wearable sensors, wireless monitoring devices, and laptops), and 7 mHealth functions (educating, communicating, goal setting, monitoring, providing feedback, reminding, and motivating). Collectively, the interventions aligned well with the concept of self-management support. However, on an individual basis (per intervention), the alignment was less strong.

**Conclusions:**

On the basis of the results, it is recommended that future research on poststroke mHealth interventions be more theoretically driven, more multidisciplinary, and larger in scale.

## Introduction

### Background

Managing the chronic effects of stroke (eg, mobility problems, cognitive impairment, and depression) has become a global health priority because of its enormous burden on health care systems [1,2]. In Canada, >400,000 people live with the effects of stroke, and by 2038, this number is expected to increase to nearly 700,000 [3]. To meet the needs of this growing population and address international priorities, self-management support interventions for stroke are of growing interest to researchers and health care professionals. Broadly defined, self-management support is a complex intervention that provides people with knowledge, confidence, and skills to manage their chronic condition [4]. Self-management support interventions have been shown to improve a variety of health outcomes after stroke, including risk factor control [5], functional ability [6], participation [6], and quality of life [7]. They have also been recommended in recent clinical practice guidelines [8]. Unfortunately, however, because of limited health care budgets and unequal access to rehabilitation, few Canadians have the opportunity to participate in self-management support interventions following stroke [8,9]. Increased access to timely, effective, and low-cost stroke self-management support could be provided through mobile health (mHealth) technology–based (eg, smartphone app–based) interventions.

Despite the growing potential, need, and interest in enhancing stroke self-management support interventions with mHealth, the evidence for its effectiveness remains unclear. In previous reviews of poststroke mHealth interventions, connections were drawn to “self-management support”; however, the concept was never explicitly defined or operationalized [10-14]. In these previous reviews, self-management support was discussed in a way that suggests that it is a newly emerging concept in the literature on poststroke mHealth interventions. Specifically, in the abstract and introduction of 3 reviews, self-management was framed as a key concept in the rationale for the review [10,12,14]. For example, in 1 review, mHealth for self-management was described as a “new strategy for stroke rehabilitation” [10]. In the discussion of 2 reviews, improved self-management was highlighted as an important outcome of mHealth use [11,13]. In the conclusion of 1 review, identifying literature on mHealth interventions to support self-management was stated as the purpose of the study [11]. Although clearly emphasizing an interest in the concept, without explicit definitions or operationalizations, the literature remains challenging to synthesize and compare, which may lead future reviews to draw incorrect conclusions about intervention effectiveness [15]. To our knowledge, no review has addressed this lack of conceptual clarity; that is, no review has aimed to map the literature on poststroke mHealth interventions according to well-accepted conceptualizations of self-management support.

### Objectives

To address this gap in the literature, we conducted a scoping review. This method was selected for its utility in clarifying key concepts in the literature, identifying key characteristics related to a concept, and identifying and analyzing knowledge gaps in an emerging field [16]. The objectives were to (1) identify and describe the types of poststroke mHealth interventions evaluated using a randomized controlled trial (RCT) design, (2) determine whether (and how) such interventions align with well-accepted conceptualizations (theory [17] and taxonomy [18]) of self-management support, and (3) identify the mHealth functions that facilitate self-management. The purpose of this study was to identify gaps in the literature and recommendations for future research related to mHealth-enhanced stroke self-management support.

## Methods

### Design

Using well-established methods [19,20], a scoping review was conducted. The protocol was not registered. A critical appraisal of the included studies was not conducted as the aim of this review was to map the breadth and depth of conceptualizations, not to draw conclusions about intervention effectiveness [16]. The PRISMA-ScR (Preferred Reporting Items for Systematic Reviews and Meta-Analyses extension for Scoping Reviews) checklist is provided in Multimedia Appendix 1 [21].

### Identifying Relevant Studies

In consultation with 2 librarians, ANT searched MEDLINE, Embase, PsycINFO, CINAHL, AMED, Scopus, and ProQuest Dissertations and Theses Global 3 times (October 2-3, 2020, February 28, 2022, and July 10, 2023). The second and third searches were conducted to identify new literature published between 2020 and 2022 and between 2022 and 2023. The search terms captured 2 search concepts: stroke and mHealth (see Multimedia Appendix 2 for the full Ovid search strategy).

### Selecting Studies

ANT, JML-M, NC, CT, VN, JR, and SJ conducted level-1 (title and abstract) and level-2 (full-text) screening in duplicate using Covidence (Veritas Health Innovation). Disagreements were resolved through consensus-based discussion. Studies were included if the article reported original research, the study included human participants with stroke or transient ischemic attack, the study evaluated an mHealth intervention (mHealth defined using 2 definitions: those of the World Health Organization—“[the] medical and public health practice supported by mobile devices, such as mobile phones, patient monitoring devices, personal digital assistants [PDAs], and other wireless devices” [22]—and Akter et al [23]—“focusing on any wireless technologies [e.g., Bluetooth, GSM, GPRS/3G, Wi-Fi, WiMAX] to transmit various health-related data content and services through mobile devices, including mobile phones, smartphones, PDAs, laptops and Tablet PCs”), and the study was an RCT. The search was limited to RCTs as a preliminary search identified a large number of studies using an RCT design. In addition, as RCTs are typically regarded as the highest in quality and presumably are the farthest along in the technology development process, their influence on research and practice was thought to be the most significant. Studies were excluded if the sample was mixed (eg, acquired brain injury), the intervention included client (person with stroke)–facing technology or equipment that was not clearly mobile and wireless, the article did not report any outcome measures related to intervention effectiveness, and the article was not written in English.

### Charting the Data

ANT developed the data-charting form in collaboration with DRD and EN. ANT charted the data verbatim and then JML-M, NC, CT, VN, JR, and SJ verified the data. Data were charted from the included articles as well as from supplementary materials and protocol papers when referenced. The data-charting form included study characteristics (eg, study aims and outcome measures), participant characteristics (eg, time since stroke and sex or gender), and intervention characteristics (based on the Template for Intervention Description and Replication checklist [24]). Visual information related to the intervention characteristics was also charted (eg, screenshots of apps).

### Collating, Summarizing, and Reporting the Results

ANT completed the data analysis in collaboration with DRD, EN, RHW, and JIC. Quantitative data were analyzed using descriptive statistics, and qualitative data were analyzed using conventional content analysis (objective 1) and directed content analysis (objectives 2 and 3) [25]. Directed content analysis for objective 2 was guided by the theory by Lorig and Holman [17] and the Practical Reviews in Self-Management Support (PRISMS) taxonomy by Pearce et al [18] as they are widely cited, slightly different conceptualizations of self-management support (see Multimedia Appendix 3 [17,18] for the operational definitions of codes). Directed content analysis for objective 3 was guided by the definition of mHealth functions by Cameron et al [26] (“the verbs describing the behavior of the system”), examples from previous research on mHealth functions [27-31], and dictionary definitions [32-38] (see Multimedia Appendix 4 [29-38] for the operational definitions of codes).

## Results

### Study Characteristics

**Figure 1:**
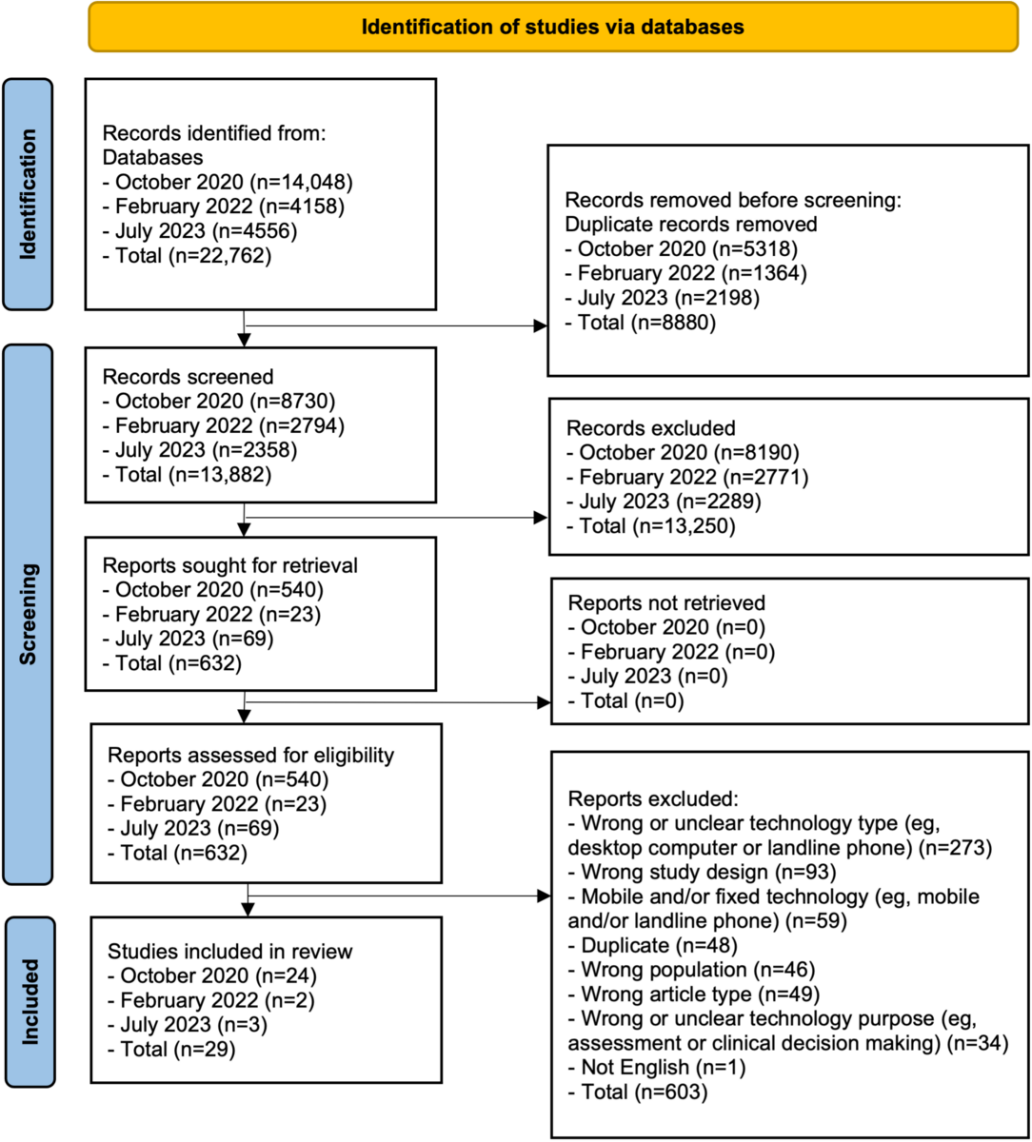
PRISMA (Preferred Reporting Items for Systematic Reviews and Meta-Analyses) flow diagram.

A total of 29 studies describing 26 interventions were included (see Figure 1 for the PRISMA [Preferred Reporting Items for Systematic Reviews and Meta-Analyses] flow diagram [39]). The studies were published between 2007 and 2023 and were from Asia (13/29, 45%), Europe (8/29, 28%), North America (4/29, 14%), Africa (2/29, 7%), and Australia (2/29, 7%). Of the 29 studies, 1 (3%) was a doctoral dissertation [40] and the remaining 28 (97%) were peer-reviewed journal articles. A total of 34% (10/29) of the studies were considered pilot, proof-of-concept, or feasibility studies. The sample sizes ranged from 11 to 4298. Table 1 presents the study and participant characteristics.

**Table 1:** Study and participant characteristics.

Study	Country	Study aims	Outcome measures	Sample size	Stroke type	Time since stroke or stroke stage	Age (y)	Sex or gender (male or men; %)
Asano et al [41]	Singapore	Effectiveness	Late-Life Function and Disability Instrument; timed 5-Meter Walk Test; 2-minute walk test; Modified Barthel Index; Activities-Specific Balance Confidence Scale; EQ-5D	98 (IG^a^: 50; CG^b^: 48)	78% ischemic; 22% hemorrhagic	Within 4 wk	Mean 64.1 (range 40.5-89.6)	52
Emmerson et al [42]	Australia	Effectiveness	Self-report logbook; Wolf Motor Function Test; customized questionnaire	58 (IG: 28; CG: 30)	86% ischemic; 15% hemorrhagic	Median 120 (range 58-226) d	Mean 66 (SD 16)	63
Ferrete Ruíz et al [43]	Spain	Effectiveness	Mississippi Aphasia Screening Test; minutes of device use	30 (IG: 23; CG: 7)	100% ischemic	Within 7 d	IG: mean 73.20 (SD 9.53); CG: mean 72.40 (SD 2.79)	50
Grau-Pellicer et al [44]	Spain	Effectiveness	Participant reports of community ambulation and sedentary behavior; 10-Meter Walk Test; 6-Minute Walk Test; Timed Up and Go test; Barthel Index; EQ-5D; satisfaction questionnaire	34 (IG: 21; CG: 13)	Not reported	IG: mean 18.92 (SD 27.6; range 1-96) mo; CG: mean 20.85 (SD 59.74; range 1-252) mo	IG: mean 62.96 (SD 11.87; range 33-89); CG: mean 68.53 (SD 11.53; range 41-83)	51
Hankinson et al [45]	Australia	Pilot study; feasibility; effectiveness	Adherence to intervention; Fugl-Meyer Assessment	15 (IG: 6; CG: 9)	Not reported	0-180 d	Not reported	59
Ifejika et al [46]	United States	Pilot; feasibility; preliminary effectiveness	Reduction in total body weight; compliance with the weight loss intervention; Patient Health Questionnaire–9; systolic blood pressure; serum low-density lipoprotein value; proportion of total hemoglobin; proportion of serum coagulation factor VIII	25 (IG: 13; CG: 12)	Not reported	Acutely hospitalized	Mean 54.1 (SD 9.4)	56
Jang and Jang [47]	South Korea	Effectiveness	Manual Muscle Test; Manual Function Test; Purdue Pegboard Test	21 (IG: 10; CG: 11)	19% ischemic; 81% hemorrhagic	Mean 963 (SD 799) d	Mean 44.5 (SD 16.5)	71
Kamal et al [48]	Pakistan	Effectiveness	Morisky Medication Adherence Scale; systolic and diastolic blood pressure; satisfaction questionnaires	162 (IG: 83; CG: 79)	84% ischemic; 17% hemorrhagic	IG: median 2 (range 1-5) mo; CG: median 2 (range 1-4) mo	IG: mean 56.07 (SD 1.5); CG: mean 57.62 (SD 1.3)	68
Kamal et al [49]	Pakistan	Effectiveness; safety	Systolic and diastolic blood pressure; blood sugar (HbA_1c_^c^); blood cholesterol (low-density lipoprotein level); mortality; modified Rankin Scale; National Institutes of Health Stroke Scale; Barthel Index	277 (IG: 141; CG: 136)	Not reported	Not reported	IG: mean 60.6 (SD 12); CG: mean 59.7 (SD 14.3)	67
Kamwesiga et al [50]	Sweden	Feasibility study; preliminary effectiveness	Logbook to record adherence; Canadian Occupational Performance Measure; self-efficacy scale; Stroke Impact Scale 3.0 Uganda version; Barthel Index; Occupational Gaps Questionnaire, Ugandan version	28 (IG: 13; CG: 15)	75% ischemic; 21% hemorrhagic; 4% unspecified	3-6 mo—IG: 10 (76.9%) and CG: 10 (66.7%); 7-11 mo—IG: 3 (23.1%) and CG: 3 (20%); 1-2 y—IG: 0 and CG: 2 (13.3%)	IG: mean 61.2 (SD 15); CG: mean 58.5 (SD 14)	25
Kang et al [51]	South Korea	Effectiveness	Regional House-Brackmann Facial Nerve Grading System; length between the corner of the mouth and the earlobe	21 (IG: 10; CG: 11)	95% ischemic; 5% hemorrhagic	Within 12 wk	IG: mean 63.1 (SD 10.3); CG: mean 55.6 (SD 16)	62
Kang et al [52]	Taiwan	Effectiveness	Stroke knowledge questionnaire; EQ-5D	63 (IG: 30; CG: 33)	43% ischemic; 57% hemorrhagic	Not reported	IG: mean 50.47 (SD 10.82); CG: mean 52.33 (SD 11.03)	68
Kenny et al [53]	United Kingdom	Feasibility; acceptability; preliminary effectiveness	Motor Status Scale; Leeds Movement Performance Index; General Self-Efficacy Scale; diary to record time spent exercising	11 (IG: 5; CG: 6)	77% ischemic; 8% hemorrhagic; 15% unspecified	Not reported	Mean 73.46 (range 41-88)	46
Kim et al [54]	South Korea	Effectiveness	Activities-Specific Balance Confidence Scale; Dynamic Gait Index; Four Square Step Test; Functional Ambulation Categories; Timed Up and Go test; up-stair and down-stair times; spatiotemporal parameters of gait (velocity and cadence)	18 (IG: 9; CG: 9)	40% ischemic; 60% hemorrhagic	IG: mean 5.68 (SD 1.04) mo; CG: mean 4.76 (SD 2.65) mo	IG: mean 58.3 (SD 11.8); CG: mean 51.8 (SD 13.7)	65
Labovitz et al [55]	United States	Effectiveness	Pill count; plasma sampling; data from artificial intelligence platform	27 (IG: 15; CG: 12)	100% ischemic	Not reported	Mean 57 (SD 13.17); median 59 (range 30-79)	46
Lakshminarayan et al [56]	United States	Pilot, proof-of-concept study; feasibility; usability; acceptability; preliminary effectiveness	Number of days blood pressure data were transmitted; systolic blood pressure; Morisky Medication Adherence Scale	50 (IG: 28; CG: 22)	Not reported	Acute	IG: mean 63.1 (SD 9.7; range 42-81); CG: mean 68.3 (SD 10.0; range 46-85); withdrawn: mean 60.33 (SD 13.7; range 47-84)	68
Maresca et al [57]	Italy	Pilot study; effectiveness	Token Test; Esame Neuropsicologico Per l’Afasia; Aphasic Depression Rating Scale; EQ-5D; Psychosocial Impact of Assistive Devices Scale	30 (IG: 15; CG: 15)	63% ischemic; 37% hemorrhagic	Not reported	Mean 51.2 (SD 11.3)	47
Moon et al [58]	South Korea	Effectiveness	Functional Dysphagia Scale; penetration-aspiration scale; visual analog satisfaction scale	16 (IG: 8; CG: 8)	88% ischemic; 13% hemorrhagic	IG: mean 22.75 (SD 9.21) d; CG: mean 21 (SD 9.02) d	IG: mean 54.13 (SD 5.41); CG: mean 55.38 (SD 14.88)	56
Øra et al [59]	Norway	Pilot study; preliminary effectiveness	Norwegian Basic Aphasia Assessment; Verb and Sentence Test; Communicative Effectiveness Index	62 (IG: 32; CG: 30)	69% ischemic; 18% hemorrhagic; 13% both	≤3 mo—IG: 16 (50%) and CG: 12 (40%); 3-12 mo—IG: 5 (15.6%) and CG: 4 (13.3%); >12 mo—IG: 11 (34.4%) and CG: 14 (46.7%)	IG: mean 64.7 (SD 11.7); CG: mean 65 (SD 12.2)	66
Pandian et al [60]	India	Effectiveness	Composite end point of recurrent stroke, high-risk transient ischemic attack, acute coronary syndrome, and all-cause mortality; change in BMI; physical activity total metabolic equivalent (min/wk); current smoking; current alcohol intake; modified Rankin Scale; medication noncompliance; systolic and diastolic blood pressure (mm Hg); fasting blood sugar (mg/dL); low-density lipoprotein cholesterol (mg/dL); triglycerides (mg/dL)	4298 (IG: 2148; CG: 2150)	83% ischemic; 17% hemorrhagic	2 d to 3 mo	IG: median 56 (range 18-88); CG: median 56 (range 18-89)	73
Radomski [40]	United States	Effectiveness	Everyday habit questionnaire; self-reported adherence to self-care checklist; Functional Independence Measure; Frenchay Activities Index; Caregiver’s Burden Scale; performance time for self-care task (seconds)	15 (IG: 5; CG 1: 5; CG 2: 5)	Not reported	Not reported	Mean 59 (SD 14)	80
Sarfo et al [61]	Ghana	Pilot; feasibility; preliminary effectiveness	Systolic and diastolic blood pressure; medication possession ratio; perceived confidence scale; Treatment Self-Regulation Questionnaire	56 (IG: 29; CG: 27)	77% ischemic; 23% hemorrhagic	<1 mo	IG: mean 54.3 (SD 11.9); CG: mean 55.9 (SD 13.7)	65
Sarfo et al [62]	Ghana	Pilot study; preliminary effectiveness	Systolic and diastolic blood pressure; medication possession ratio score; Morisky Medication Adherence Scale; perceived confidence scale; Treatment Self-Regulation Questionnaire; Telemedicine Satisfaction and Usefulness Questionnaire; hypertension and stroke knowledge 14-item questionnaire	55 (IG: 28; CG: 27)	77% ischemic; 23% hemorrhagic	<1 mo	IG: mean 54.3 (SD 11.9); CG: mean 55.9 (SD 13.7)	65
Tomori et al [63]	Japan	Pilot study; feasibility; preliminary effectiveness	36-Item Short Form Health Survey; Brunnstrom recovery stages; Functional Independence Measure; Client Satisfaction Questionnaire; duration of stay	37 (IG: 16; CG: 21)	Not reported	≥30 d; subacute	Mean 66.22 (SD 10.64)	67
Vahlberg et al [64]	Sweden	Effectiveness	6-Minute Walk Test (m); chair stand test (s); 10-Meter Walk Test (m/s); Short Physical Performance Battery	79 (IG: 40; CG: 39)	72% ischemic; 11% hemorrhagic; 17% transient ischemic attack	Mean 6 (SD 4.4) d	IG: mean 63.9 (SD 10.1); CG: mean 63.9 (SD 10.8)	63
Vahlberg et al [65]	Sweden	Effectiveness	Fat-free mass (kg); fat mass (kg); BMI; body weight (kg); HbA_1c_; serum insulin-like growth factor; low- and high-density lipoprotein cholesterol; self-reported health; mortality	71 (IG: 36; CG: 35)	72% ischemic; 11% hemorrhagic; 17% transient ischemic attack	Median 5 d	IG: mean 63.9 (SD 10); CG: mean 63.9 (SD 10)	63
Wan et al [66]	China	Effectiveness	Systolic and diastolic blood pressure; Health-Promoting Lifestyle Profile II	158 (IG: 80; CG: 78)	100% ischemic	Within 1 mo	Median 63.81	65
Wang et al [67]	China	Effectiveness	Health-Promoting Lifestyle Profile II; systolic and diastolic blood pressure; modified Rankin Scale; stroke recurrence	151 (IG: 76; CG: 75)	100% ischemic	Within 1 mo	Median 63.80	66
Wang et al [68]	China	Effectiveness	Systolic blood pressure; Self-Management Ability Scale; Morisky Medication Adherence Scale; BMI; blood low-density lipoprotein	193 (IG: 98; CG: 95)	67% ischemic; 33% hemorrhagic	Not reported	IG: mean 42.75 (SD 0.16); CG: mean 41.32 (SD 2.16)	61

^a^IG: intervention group.

^b^CG: control group.

^c^HbA_1c_: glycated hemoglobin.

### Participant Characteristics

In total, 62% (18/29) of the studies included participants with both ischemic and hemorrhagic stroke, and 7% (2/29) also included transient ischemic attack. Of the 23 studies that reported participants’ stroke stage or time since stroke, 16 (70%) focused on the subacute stage (7 d to 6 mo after stroke). The average age of the participants ranged from 42 to 74 years (weighted average 57, weighted SD 4.46). No studies differentiated between sex and gender. A total of 83% (24/29) of the studies included more male participants or men than female participants or women, ranging from 25% to 80% of male participants or men. Some studies reported on participants’ education (15/29, 52%), marital status (8/29, 28%), employment status (6/29, 21%), and geographic location (5/29, 17%), and fewer studies reported on race (3/29, 10%), ethnicity (1/29, 3%), and income (2/29, 7%).

### Objective 1: Types of Poststroke mHealth Interventions

Multimedia Appendix 5 [40-68] summarizes the interventions individually and the following sections summarize the interventions collectively, according to selected items from the Template for Intervention Description and Replication checklist [24].

#### Why: Describe Any Rationale, Theory, or Goal of the Elements Essential to the Intervention

mHealth technology was rationalized as a strategy to improve intervention effectiveness (18/26, 69%), access (13/26, 50%), convenience (6/26, 23%), and cost-effectiveness (5/26, 19%). A total of 5 interventions were explicitly based on a theory, model, framework, or taxonomy: self-determination theory (n=2, 40%); Health Belief Model (n=2, 40%); social cognitive theory (n=1, 20%); the International Classification of Functioning, Disability, and Health framework (n=1, 20%); the Coventry, Aberdeen, and London–Refined taxonomy of behavior change techniques (n=1, 20%); and a proposed ecological model of adherence to rehabilitation treatment recommendations (n=1, 20%). Common goals of the interventions were to improve outcomes related to treatment or medication adherence (10/26, 38%), motor or physical activity (8/26, 31%), functional ability or independence (5/26, 19%), speech, language, or swallowing (5/26, 19%), hypertension or blood pressure control (5/26, 19%), risk factor control (5/26, 19%), and quality of life (3/26, 12%).

#### What: Describe Any Physical or Informational Materials Used and Each of the Procedures, Activities, or Processes Used in the Intervention

A total of 7 focal areas were identified: physical exercise (10/26, 38%), risk factor management (5/26, 19%), linguistic exercise (3/26, 12%), activities of daily living (ADLs) training (3/26, 12%), medication adherence (2/26, 8%), stroke education (2/26, 8%), and weight management (1/26, 4%). In total, 5 types of mobile devices were used: mobile phones or smartphones (17/26, 65%), tablets (9/26, 35%), wearable sensors (5/26, 19%; eg, pedometers or wearable bracelets), wireless monitoring devices (4/26, 15%; eg, Bluetooth sphygmomanometers or Bluetooth blood glucose meters), and laptops (1/26, 4%). Within devices, the features used included: apps (15/26, 58%), messaging (12/26, 46%; eg, via an app or SMS text messaging), phone calling (7/26, 27%), videos (6/26, 23%), videoconferencing (3/26, 12%), and email (2/26, 8%). All but 4 interventions (22/26, 85%) were self-directed, and 8% (2/26) were gamified.

#### Who Provided: For Each Category of Intervention Provider, Describe Their Background

The interventions were provided by researchers (9/26, 35%), occupational therapists (7/26, 27%), physical therapists (4/26, 15%), nurses (4/26, 15%), speech-language pathologists (2/26, 8%), physicians (2/26, 8%), pharmacists (1/26, 4%), neuropsychologists (1/26, 4%), brain and heart health managers (1/26, 4%), allied health professionals (1/26, 4%), clinicians (1/26, 4%), and clinic staff (1/26, 4%). In total, 12% (3/26) were provided by a multidisciplinary team of health care professionals.

#### How: Describe the Modes of Delivery of the Intervention and Whether It Was Provided Individually or in a Group

A total of 85% (22/26) of the interventions were delivered both virtually (eg, via videoconferencing or SMS text messaging) and in-person (eg, in-person orientation or clinic visits). In total, 77% (20/26) were individual based (delivered to the individual with stroke), 38% (10/26) were dyad based (delivered to the individual with stroke and their caregiver or family member), and 8% (2/26) were group based (delivered to groups of people with stroke).

#### Where: Describe the Types of Locations Where the Intervention Occurred

In total, 58% (15/26) of the interventions occurred both at the hospital or clinic (in-person component) and the participants’ home (virtual component).

#### When and How Much: Describe the Number of Times the Intervention Was Delivered and Over What Period

Intervention delivery time ranged from 14 days to 1 year, with the most common being 4 weeks (5/26, 19%) and 6 months (5/26, 19%). Session frequency varied (twice/d to once every 2-3 mo), as did session length (5 min to 1 h). This variability reflects a wide range of session types (eg, exercise sessions, education sessions, blood pressure self-monitoring, and clinic visits). There was also variability in the dosage of technology used, such as the schedule for sending and receiving messages (twice/d to once/wk) and the amount of time connected to the devices (eg, 1 intervention required participants to wear a pedometer at all times except when sleeping, bathing, or swimming).

#### Tailoring: If the Intervention Was Planned to be Personalized, Titrated, or Adapted, Describe What, Why, When, and How

A total of 69% (18/26) of the interventions involved tailoring to the person with stroke (eg, abilities, goals, or preferred music). In total, 8% (2/26) of the interventions involved tailoring to the caregiver or family member (eg, preferred ADLs) [40,50]. A total of 12% (3/26) of the interventions involved self-tailoring by the person with stroke (eg, education topics [52] or exercises [53,64,65]).

### Objective 2: Alignment With Self-Management Support Theory and Taxonomy

**Figure 2:**
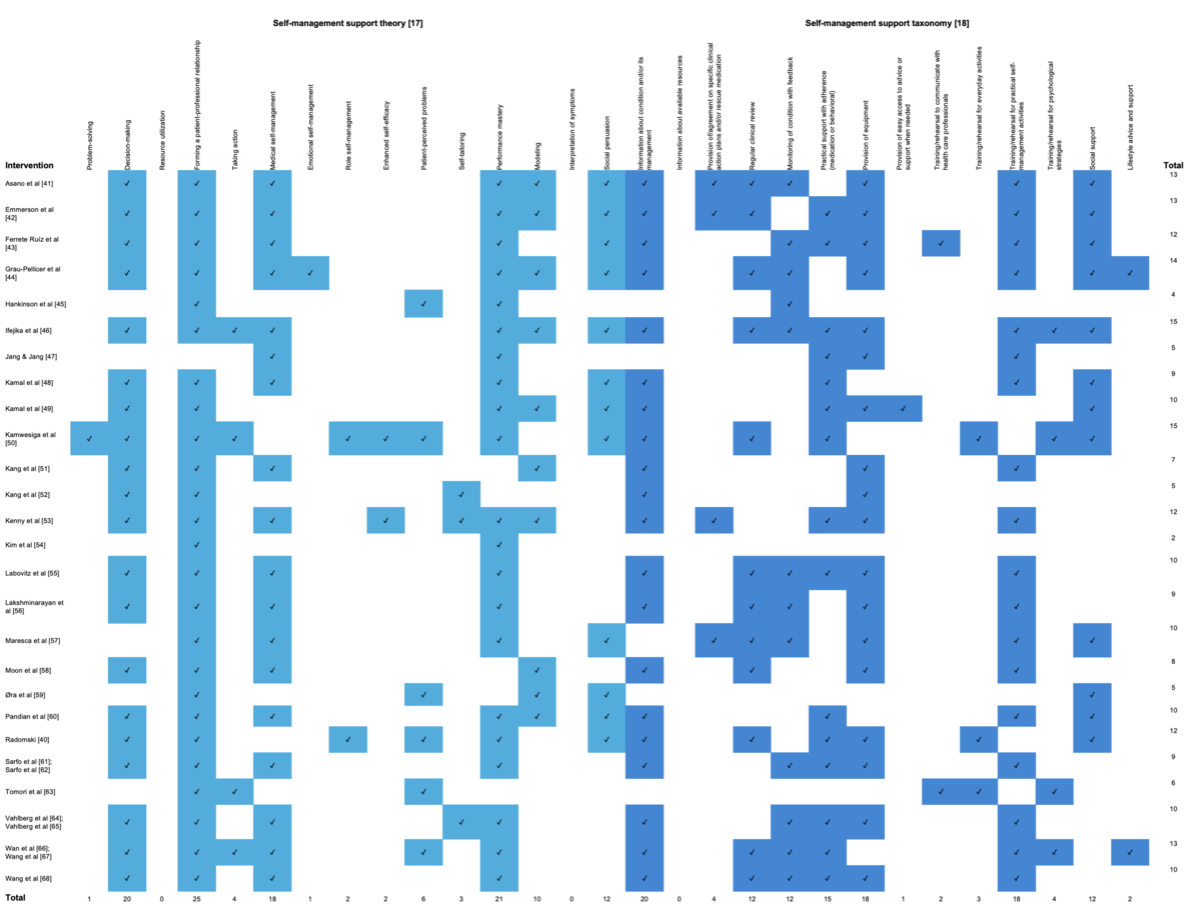
Extent and range of alignment between poststroke mobile technology–based interventions and self-management support theory and taxonomy. Please see Multimedia Appendix 6 for a larger version.

Of the 29 conceptual variables, 26 (90%) were coded at least once. The number of interventions coded per variable ranged from 0 to 25 (mean 8.55). The number of variables coded per intervention ranged from 2 to 15 (mean 9.54). Figure 2 [17,18,40-68] presents the extent and range of alignment, and Table 2 presents the nature of alignment.

**Table 2:** Nature of alignment between poststroke mobile technology–based interventions and self-management support theory and taxonomy.

Conceptual variable						Intervention examples
**Self-management support theory [17]**						
	**Core self-management skills**					
		Problem-solving			Clients developed skills in problem-solving through training focused on the Target-Plan-Perform-Prove strategy [50].	
		Decision-making			Clients developed skills in decision-making through learning information about stroke, rehabilitation, medications, lifestyle, and risk factors (eg, stroke history, heart disease, atrial fibrillation, obesity, age, sleep patterns, diet, exercise, and blood pressure [40,46,48-50,52,60-62,66-68]), as well as by learning how to perform exercises [41-44,51,53,58,60,64,65], take medications [55], measure blood pressure [56,61,62,66-68], and measure blood glucose [68].	
		Resource utilization			Not reported	
		Forming a patient-professional relationship			Clients developed skills in forming a relationship with a professional through participating in virtual (eg, videoconference or phone call) and in-person sessions [40-46,48-60,63,66-68], as well as by sending and receiving messages (eg, via an app or SMS text messaging [40,44,48,50,60-62,64-68]).	
		Taking action			Clients developed skills in taking action through goal-setting training focused on weight loss [46], ADLs^a^ [50,63], and self-management (eg, medication adherence [66,67]).	
	**Self-management tasks or behaviors**					
		Medical self-management			Clients practiced the medical tasks of performing self-directed physical or linguistic exercises [41-44,47,51,53,57,58,60,64,65], taking medications [48,55], and self-monitoring physical health data (weight, diet, and exercise [46,68]; sleep [68]; fatigue [44]; blood pressure [56,61,62,66-68]; and blood glucose [68]).	
		Emotional self-management			Clients practiced the emotional task of self-monitoring mood through app-based questionnaires [44].	
		Role self-management			Clients practiced maintaining or changing behaviors related to ADLs or life roles (eg, dressing or cooking) in the context of their home environment [40,50].	
	**Mechanism of change**					
		Enhanced self-efficacy			Self-efficacy was used as an outcome measure [50,53].	
	**Characteristics of self-management support**					
		Patient-perceived problems			Interventions were based on client-identified problems, concerns, or goals [40,45,50,59,63,66,67].	
		Self-tailoring			Self-tailoring was encouraged by instructing clients to select their own educational content based on their time and needs [52], practice the exercises as often as they wished [53], and gradually modify the exercises based on preference and perceived strenuous intensity [64,65].	
		**Efficacy enhancement**				
			Performance mastery	Performance mastery was promoted through feedback (eg, via an app or wearable sensor, through SMS text messaging, or from a therapist [40-47,49,54-57,61,62,64,65,68]) and self-reflection (eg, through checklists or diaries or from reminder messages prompting a response [40,43,47-50,53,60,64-67]).		
			Modeling	Modeling was offered through group-based formats [44,59]; demonstration videos (eg, of the therapist or client performing the exercises or of animated characters taking medications [41,42,49,53,58]); fictional and nonfictional stories about people with stroke that are age appropriate, country specific, and culturally relevant (eg, Mahatma Gandhi was a character in a story created for an Indian audience [60]); distorted mirror reflections via an app that allowed clients to watch the reflection of the unaffected half of their face as if it were the affected half [51]; and culturally competent counseling by clinicians of clients’ ethnic groups [46].		
			Interpretation of symptoms	Not reported		
			Social persuasion	Social persuasion was promoted through group-based (eg, a WhatsApp group was created for clients to motivate each other to maintain an active lifestyle [44,59]) and dyad (client-caregiver or family member)-based (eg, caregivers were instructed to support the client at home in using the technology and adhering to the program [40-43,46,48-50,57,60]) formats.		
**Self-management support taxonomy [18]**						
	Information about condition and/or its management				Clients were provided with general information about stroke, rehabilitation, medications, lifestyle, and risk factors (eg, stroke history, heart disease, atrial fibrillation, obesity, age, sleep patterns, diet, exercise, and blood pressure [40,46,48-50,52,60-62,66-68]) as well as general instruction on how to perform exercises [41-44,51,53,58,60,64,65], take medications [55], measure blood pressure [56,61,62,66-68], and measure blood glucose [68].	
	Information about available resources				Not reported	
	Provision of or agreement on specific clinical action plans and/or rescue medication				Clients were provided with specific, tailored instruction on how to perform physical or linguistic exercises [41,42,53,57].	
	Regular clinical review				Clients connected with health care professionals on a regular basis for a scheduled review of their condition and self-management (eg, via videoconference, phone call, messaging, or in-person sessions [40-42,44,46,50,55-58,66-68]).	
	Monitoring of condition with feedback				Clients monitored symptoms, behaviors, or objective measures related to their condition (eg, exercise adherence data from wearable sensors, weight management data through tracking calories consumed, medication adherence data via app-based artificial intelligence, and blood pressure data from wireless monitoring devices [41,43-46,55-57,61,62,64-68]). Professionals also reviewed the monitored data and provided clients with feedback [41,44,55-57,61,62,68].	
	Practical support with adherence (medication or behavioral)				Practical support with adherence was provided to clients in the form of reminder alarms [42], reminder messages (eg, via push notifications within an app or through SMS text messaging [40,46,48-50,55,61,62,64-68]), reminder phone calls [48,50,60], and reminder sheets (eg, checklists, diaries, and calendars [40,43,47,50,53,60,64-67]).	
	Provision of equipment				Self-monitoring or self-management was enabled, assisted, or promoted through the provision of tablets [41-43,47,49,51,53,57], smartphones [40,56,58,61,62], wearable sensors (eg, pedometers or wearable bracelets [41,44,64,65,68]), wireless monitoring devices (eg, Bluetooth sphygmomanometers or Bluetooth blood glucose meters [41,56,61,62,68]), apps [43,44,46,47,49,51,52,55,56,61,62,68], and measuring cups [46].	
	Provision of easy access to advice or support when needed				Clients were provided with a 24/7 stroke helpline number [49].	
	Training or rehearsal to communicate with health care professionals				Clients developed communication skills by using apps to communicate their needs with nursing staff [43] and participate in shared decision-making with occupational therapists for goal setting [63].	
	Training or rehearsal for everyday activities				Clients developed skills to support ADLs (eg, dressing, cooking, or knitting [40,50,63]).	
	Training or rehearsal for practical self-management activities				Clients developed specific, practical skills in performing self-directed physical or linguistic exercises [41-44,47,51,53,57,58,60,64,65], taking medications [48,55], and self-monitoring health data (weight, diet, and exercise [46,68]; sleep [68]; fatigue [44]; blood pressure [56,61,62,66-68]; blood glucose [68]; and mood [44]).	
	Training or rehearsal for psychological strategies				Clients developed psychological skills in goal setting [46,50,63,66,67] and problem-solving [50].	
	Social support				Social support was facilitated through group-based (eg, a WhatsApp group was created for clients to facilitate peer support [44,59]) and dyad (client-caregiver or family member)-based (eg, caregivers were encouraged to watch educational videos with the client and engage in a follow-up discussion with a professional afterward or instructed to support the client at home in using the technology and adhering to the program [40-43,46,48-50,57,60]) formats.	
	Lifestyle advice and support				Lifestyle advice and support were provided to clients through a peer-based WhatsApp group [44] and nurse-led education and goal-setting sessions [66,67].	

^a^ADL: activity of daily living.

### Objective 3: mHealth Functions That Facilitate Self-Management

Across all conceptual variables and interventions, 7 mHealth functions were identified as facilitating self-management: educating, communicating, goal setting, monitoring, providing feedback, reminding, and motivating.

## Discussion

### Principal Findings

#### Overview

To our knowledge, this is the first scoping review to map the literature on poststroke mHealth interventions according to a self-management support theory and taxonomy. A total of 29 studies describing 26 interventions were included. Overall, we found that the interventions addressed 7 focal areas, 5 types of mobile devices, and 7 mHealth functions. Collectively, the interventions aligned well with the concept of self-management support. However, on an individual basis (per intervention), the alignment was less strong. The following sections further explain how this review extends previous reviews on poststroke mHealth [10-14,69-72] and telehealth [73] interventions in relation to the study objectives and interventions included.

#### Objective 1: Types of Poststroke mHealth Interventions

##### Focal Areas: Current Trends and Gaps

Our first objective was to identify and describe the types of poststroke mHealth interventions evaluated using an RCT design. Speaking to such types, 7 focal areas were identified: physical exercise, risk factor management, linguistic exercise, ADLs training, medication adherence, stroke education, and weight management. These 7 focal areas have been identified in previous reviews on poststroke mHealth interventions [10-14,69-72]; however, the included interventions varied. Compared with previous reviews, 45% (13/29) of the studies included in our review (12 interventions) had not been previously identified. Similar to previous reviews, this review found the most common focal area to be physical exercise, likely reflecting the rising trend within the general population of using mobile technology to promote physical fitness in everyday life [74,75]. Hence, the literature clearly supports continued research on poststroke mHealth interventions for physical exercise. Also consistent with previous reviews, this review did not identify any interventions focused on mood or fatigue. Considering the high prevalence of poststroke depression, anxiety, and fatigue, this is a serious gap that should be addressed in future research [76]. Surprisingly, unlike 5 previous reviews [10,11,13,70,71], this review did not identify any interventions focused on cognition. This difference was due to the varying eligibility criteria (eg, study design). Given this difference across reviews as well as the high prevalence of poststroke cognitive impairment [76], future research on poststroke mHealth interventions for cognition is encouraged to progress toward the level of RCTs.

##### mHealth Technology: Positioning on the Spectrum of Definitions

Regarding the types of technology used in the interventions, our review identified 5 types of mobile devices (mobile phones or smartphones, tablets, wearable sensors, wireless monitoring devices, and laptops) and 6 features within these devices (apps, messaging, phone calling, videos, videoconferencing, and email). This wide range of technologies resulted from our novel approach to defining mHealth. Previous reviews on poststroke mHealth interventions have defined mHealth either very narrowly, focusing on a few specific mobile devices or features (eg, mobile phones [72], wearable activity monitors [69], or mobile apps for phones [10,11,14,69,71] and tablets [10,11,14,70,71]), or very broadly, focusing on mHealth in general and including devices and features that may not be entirely mobile and wireless (eg, computer programs [10,12,71], telephone calls [12], and web-based applications [13]). Our review was interested in the literature between these 2 ends of the narrow-broad spectrum of mHealth definitions. We followed the recommendation of Cameron et al [26] to define mHealth in a way that captures the “combinatorial complexity” of the mobile system and used 2 open-ended definitions of mHealth [22,23]. Thus, we captured additional literature on poststroke mHealth interventions by focusing on entirely mobile *systems* (technology and equipment) of any type (devices and features). As the field of mHealth continues to grow, we suggest that future reviews explicitly position themselves on this narrow-broad spectrum of mHealth definitions so that the literature can be more readily interpreted and applied. In addition, future work should build on that by Cameron et al [26] to further deepen our understanding of the mobile system.

#### Objective 2: Alignment With Self-Management Support Theory and Taxonomy

Our second objective was to determine whether (and how) the included interventions aligned with well-accepted conceptualizations (theory [17] and taxonomy [18]) of self-management support. Collectively, the interventions addressed 90% (26/29) of the conceptual variables, whereas individual interventions only addressed an average of 33% (9.54/29) of the conceptual variables. This discrepancy speaks to the potential for improvements in the alignment between poststroke mHealth interventions and the concept of self-management support. The results also revealed key conceptual variables missing from the literature, such as “emotional self-management” and “information about available resources.” Hence, the results suggest that future research should be more closely aligned with the theory and taxonomy of self-management support. Previous reviews on poststroke mHealth interventions [10-14,69-72] have not mapped the literature in this way. However, a review of poststroke telehealth interventions [73] used the PRISMS taxonomy [18] in a similar way, further validating the relevance of this approach.

#### Objective 3: mHealth Functions That Facilitate Self-Management

Our third objective was to identify the mHealth functions that facilitate self-management. A total of 7 mHealth functions were identified: educating, communicating, goal setting, monitoring, providing feedback, reminding, and motivating. These 7 functions, although together framed as facilitating self-management, are not inherently specific to self-management support interventions as they speak generally to what the intervention *does*, not specifically to what the intervention is *about*. Viewing mHealth functions in this way, as generic “verbs describing the behavior of the system” [26] or as action words that link technology capabilities with intervention components, has not been done in past reviews on poststroke mHealth interventions [10-14,69-72]. However, this approach to conceptualizing mHealth functions does align with other work in the broader field of mHealth [26,30]. Future research is encouraged to build on this approach and use the identified functions to describe how specific technology capabilities are linked to specific intervention components. Specifically linking technology capabilities with intervention components is important as it would allow for more systematic examinations as to what it is about delivery through mHealth that may be superior or not to other intervention delivery modalities (eg, is educating on sensitive topics via mHealth better than via in-person groups?).

### Recommendations for Future Research

The purpose of this study was to identify gaps in the literature and recommendations for future research related to mHealth-enhanced stroke self-management support. In total, 3 overarching recommendations for future research were identified. First, future research should be more explicit about the theories their interventions are based on as well as their conceptualizations of self-management support. Using theory and other conceptualizations in this way would help promote a common language of self-management support and ensure that all conceptual variables are considered, which could ultimately improve intervention adherence, effectiveness, replicability, and uptake in clinical practice. Second, future research should be more multidisciplinary so that a wider range of conceptual variables can be addressed per intervention. This multidisciplinary approach to improving alignment would likely lead to more comprehensive, holistic, and effective interventions. Third, future research should use larger sample sizes and consider using pragmatic trial designs to establish real-world effectiveness.

### Limitations

The search was limited to the English language, so the findings may be biased toward English-speaking countries, although 15 countries were represented. Directed content analysis, as with any qualitative approach, involves subjectivity; to address this, operational definitions for codes were used and reported. Finally, this review focused on RCTs, so the findings may be biased toward more traditional or RCT-suited interventions. Given the challenges associated with conducting RCTs on technology-based interventions [77], future reviews should consider including other study designs.

### Conclusions

This scoping review clarified the concept of self-management support in the literature on poststroke mHealth interventions by mapping studies according to well-accepted conceptualizations of self-management support. On the basis of the results, it is recommended that future research on poststroke mHealth interventions be more theoretically driven, more multidisciplinary, and larger in scale.

## Appendix

Multimedia Appendix 1 PRISMA-ScR (Preferred Reporting Items for Systematic Reviews and Meta-Analyses extension for Scoping Reviews) checklist.

Multimedia Appendix 2 Search strategy for the Ovid databases (MEDLINE, Embase, PsycINFO, and AMED).

Multimedia Appendix 3 Operational definitions used in the directed content analysis related to self-management support theory and taxonomy (objective 2).

Multimedia Appendix 4 Operational definitions used in the directed content analysis related to mobile health functions (objective 3).

Multimedia Appendix 5 Intervention characteristics according to selected items from the Template for Intervention Description and Replication checklist.

Multimedia Appendix 6 Extent and range of alignment between poststroke mobile technology–based interventions and self-management support theory and taxonomy.

## Abbreviations

ADL: activity of daily living
mHealth: mobile health
PRISMA: Preferred Reporting Items for Systematic Reviews and Meta-Analyses
PRISMA-ScR: Preferred Reporting Items for Systematic Reviews and Meta-Analyses extension for Scoping Reviews
PRISMS: Practical Reviews in Self-Management Support
RCT: randomized controlled trial
