# Supplementary material for: Mobile Technology–Based Interventions for Stroke Self-Management Support: Scoping Review
Source: JMIR Mhealth Uhealth. 2023 Dec 6;11:e46558. doi: 10.2196/46558 (PMC10733834; doi:10.2196/46558)
Supplement: Multimedia Appendix 2 [file mhealth_v11i1e46558_app2.docx]

**Multimedia Appendix 2.** Search strategy for the Ovid databases (MEDLINE, Embase, PsycINFO, and AMED).

**Used on October 2, 2020**

| **#** | **Search** |
| --- | --- |
| 1 | stroke/ or brain infarction/ or stroke, lacunar/ |
| 2 | (stroke or brain infarct* or lacunar infarct* or cerebrovascular accident or CVA).ti,ab,kf. |
| 3 | ((cerebr* or brain) adj3 infarct*).ti,ab,kf. |
| 4 | 1 or 2 or 3 |
| 5 | telecommunications/ or telemedicine/ or telerehabilitation/ or telemetry/ |
| 6 | (telecommunications or telemetry or telemedicine or tele-medicine or telehealth* or tele-health or telestroke or tele-stroke or telerehab* or tele-rehab* or teletherap* or tele-therap* or electronic health or eHealth or e-health or mobile health or mHealth or m-health or digital health*).ti,ab,kf. |
| 7 | remote sensing technology/ or cell phone/ or text messaging/ or wireless technology/ or computers, handheld/ or smartphone/ or mobile applications/ |
| 8 | (remote sensing technolog* or cell phone* or cellular phone* or mobile device* or mobile app* or mobile phone* or smartphone* or smart phone* or text messag* or texting or sms* or short messag* or mms* or multimedia messag* or multi-media messag* or wireless technolog* or mobile computer* or handheld computer* or hand-held computer* or tablet* or personal digital assistant* or PDA or ipad or iphone or galaxy tab or surface pro or palm pilot).ti,ab,kf. |
| 9 | 5 or 6 or 7 or 8 |
| 10 | 4 and 9 |
| 11 | Limit 10 to English language |

**Used on February 28, 2022**

| **#** | **Search** |
| --- | --- |
| 12 | (20201* or 2021* or 2022*).dt,ez,ed. |
| 13 | 11 and 12 |

**Used on July 10, 2023**

| **#** | **Search** |
| --- | --- |
| 12 | (2022* or 2023*).dt,ez,ed. |
| 13 | 11 and 12 |
