# Supplementary material for: Mobile Technology–Based Interventions for Stroke Self-Management Support: Scoping Review
Source: JMIR Mhealth Uhealth. 2023 Dec 6;11:e46558. doi: 10.2196/46558 (PMC10733834; doi:10.2196/46558)
Supplement: Multimedia Appendix 3 [file mhealth_v11i1e46558_app3.docx]

**Multimedia Appendix 3.** Operational definitions used in the directed content analysis related to self-management support theory and taxonomy (objective 2).

| **Code** | | | **Operational Definition** |
| --- | --- | --- | --- |
| **Core self-management skills** | **Problem solving** | | “This does not mean that people are taught solutions to their problems. Rather, they are taught basic problem-solving skills. These include problem definition, generation of possible solutions including the solicitation of suggestions from friends and health care professionals, solution implementation, and evaluation of results” [17 p.2].  *Update added during data analysis: Coding requires explicit mention of engaging the client in formal problem-solving training.* |
|  | **Decision making** | | “When this decision making is part of problem solving, it is part of the D’Zurilla model. In addition, persons with chronic illness must make day-to-day decisions in response to changes in disease condition. To do this they must have the knowledge necessary to meet common changes. For example, how do I know when I have exercised enough or too much? How do I know whether a symptom is medically serious? Should I continue taking my medications when I have a fever? What do I do to get back on my diet if I eat some chocolate cake? Decision making is based on having enough and appropriate information” [17 p.2].  *Update added during data analysis: Coding requires explicit mention of providing clients with information related to self-management. Information should help clients feel actively involved in their care. If the information arises from experiential learning, it should be actively not passively processed, such that it could be reasonably assumed that the information could be applied in different contexts (clients should be able to recall/apply the information they learned).* |
|  | **Resource utilization** | | “Many programs tell participants about resources but do not teach participants how to use the phone book, 800 numbers, the Internet, the library, and community resource guides. In addition to teaching people how to use resources, self-management includes helping people seek these out from many sources” [17 p.2].  *Update added during data analysis: Coding requires explicit mention of engaging the client in resource utilization/navigation training.* |
|  | **Forming a patient/**  **professional relationship** | | “When dealing with a long-term illness, the role of the health care provider becomes that of teacher and partner as well as professional supervisor. The patient must be able to report accurately the trends and tempo of the disease, make informed choices about treatment, and discuss  these with the health care provider” [17 p.2-3].  *Update added during data analysis: Coding requires a clear suggestion of relationship building/ongoing communication between the client and a professional (interactions must be suggested to go beyond basic assistance with technology set-up/installation).* |
|  | **Taking action** | | “The final skill is taking action. This can be acquainted with solution implementation in the D’Zurilla model and with skill mastery in the self-efficacy model. Taking action may seem more like a decision than a skill but, in fact, there are skills involved in learning how to change a behavior. The most important of these is probably making a short-term action plan and carrying it out. … An action plan involves a period of 1 or 2 weeks and is very behavior specific” [17 p.3].  *Update added during data analysis: Coding requires explicit mention of engaging the client in formal goal setting.* |
| **Self-management**  **tasks/**  **behaviours** | **Medical self-management** | | “The first set of tasks involves the medical management of the condition such as taking medication, adhering to a special diet, or using an inhaler” [17 p.1].  *Update added during data analysis: Coding requires explicit mention of engaging the client in self-management behaviours/activities/training related to physical and/or cognitive health.* |
|  | **Emotional self-management** | | “The [second] task requires one to deal with the emotional sequeli of having a chronic condition, which alters one’s view of the future. Emotions such as anger, fear, frustration, and depression are commonly experienced by someone with a chronic disease; therefore, learning to manage these emotions becomes part of the work required to manage the condition” [17 p.1].  *Update added during data analysis: Coding requires explicit mention of engaging the client in self-management behaviours/activities/training related to mental health.* |
|  | **Role self-management** | | “The [third] set of tasks involves maintaining, changing, and creating new meaningful behaviors or life roles. For example, people with back pain may need to change the way they garden or participate in favorite sports. For someone with heart or pulmonary disease it may mean doing less, such as cooking only one dish for a holiday dinner while other people prepare the other dishes” [17 p.1].    *Update added during data analysis: Coding requires explicit mention of engaging the client in self-management behaviours/activities/training related to activities of daily living.* |
| **Mechanism of change** | **Enhanced self-efficacy** | | “Perceived self-efficacy refers to beliefs in one’s capabilities to organize and execute the courses of actions required to produce given attainments … We have shown that both base-line self-efficacy and changes in self-efficacy are associated with future health status … Thus, it appears that enhanced self-efficacy is at least one of the mechanisms responsible for the improvements in health status demonstrated by those attending self-management programs” [17 p.4].  *Update added during data analysis: Coding requires self-efficacy to be an outcome measure.* |
| **Additional**  **characteristics of self-management**  **programs** | **Patient perceived problems** | | “Self-management programs must be based on patient perceived problems. For example, traditional arthritis education programs focus on preventing disability and disability management. However, the major concern of arthritis patients is pain … Therefore, arthritis self-management programs focus on pain management. This does not mean that information about managing disability is not taught. Rather, it is taught in the context of pain management. Because self-management education is focused on patient concerns and problems, a detailed needs assessment must be  done for each new topic and group of patients” [17 p.2].  *Update added during data analysis: Coding requires a clear suggestion that the intervention was based on client-identified/perceived problems/concerns and/or goals.* |
|  | **Self-tailoring** | | “[Another] characteristic distinguishes self-management from more traditional health promotion and patient education programs: self-tailoring, or using self-management skills and knowledge and applying these to oneself as appropriate” [17 p.3].  *Update added during data analysis: Coding requires explicit mention of engaging the client in self-tailoring.* |
|  | **Efficacy enhancement** | **Performance mastery** | “Skills mastery or taking action involves getting people actively involved in behavior change. It is very difficult for someone to argue that they cannot do something when they are doing it. In our courses, action planning is the key element in skills mastery” [17 p.4].    *Update added during data analysis: Coding requires explicit mention of engaging the client in behaviour change and a clear suggestion that there was an opportunity for the client to reflect on their behaviour change and gain a sense of mastery.* |
|  |  | **Modelling** | “Modeling can be accomplished several ways. First, materials (written and video) should reflect the population for which they are developed. Thus, drawings, photos, and actors should represent various body types, ages, and races as well as both sexes. … Modeling can also be accomplished by having peers teach self-management programs. … Finally, people can act as models for each other. In group situations, when a patient has a problem, other members of the group can be asked to offer suggestions before any are offered by the group leader. In much this same way, people newly diagnosed with a disease, or contemplating a new treatment, can be paired with someone who has experience with the disease or treatment” [17 p.4].    *Update added during data analysis: Coding requires explicit mention of providing clients with any sort of relevant (social) model (virtual/in-person, live/pre-recorded etc.) to compare their behaviour to/learn from (can be themselves, a peer, a professional, and/or a fictional character/actor). It will be assumed that group-based interventions will offer this unless otherwise stated. A professional will be considered a “relevant” model when they clearly possess a unique characteristic which makes them especially relevant to the clients (e.g., from the same ethnic group).* |
|  |  | **Interpretation**  **of symptoms** | “Reinterpreting physiologic symptoms is aimed at helping patients form alternative explanations for their symptoms. When they have alternative explanations as to cause, they also have reasons to try new self-management behaviors. For this reason, symptoms should be explained as having multiple causes. This leads to multiple ways of managing the symptom” [17 p.4].    *Update added during data analysis: Coding requires explicit mention of providing clients with information on or engaging in a discussion around the interpretation of symptoms.* |
|  |  | **Social persuasion** | “Social persuasion is a powerful means of increasing self-efficacy. If those around you are participating in a behavior or not participating in a behavior, you are more likely to follow” [17 p.4].    *Update added during data analysis: Coding requires explicit mention of other people being involved/connected with the client throughout the intervention (besides the professional delivering the intervention). It will be assumed that dyad- and group-based interventions will offer this unless otherwise stated.* |
| **Practical Reviews in Self-Management Support (PRISMS) taxonomy of self-management support** | **Information about**  **condition and/or its**  **management** | | “Providing people with long-term conditions with information and instruction about their condition or about general aspects and principles of managing their condition (physiology, medication, prognosis, emotional, psychosocial, etc.)” [18 p.78].  “Examples of activities from PRISMS dataset … Explanation of anatomy, pulmonary physiology, pathophysiology of lung and factors that can provoke asthma (Asthma). Two day patient education programme, covering living with epilepsy, epidemiology, basic knowledge, diagnostics, therapy, self-control, prognosis, psychosocial aspects and network (Epilepsy)” [18 p.78]. |
|  | **Information about available resources** | | “Providing people with long-term conditions with information (e.g. written, verbal, visual) on issues such as financial benefits, sources of social or peer support, charitable organizations” [18 p.78].  “Examples of activities from PRISMS dataset … Advice on obtaining financial assistance and transport (Stroke). Referral to benefits advisor to ensure individual is in receipt of benefits to which they are entitled (COPD)” [18 p.78]. |
|  | **Provision of/agreement on specific clinical action plans and/or rescue medication** | | “Written instructions prepared with or by a healthcare professional to enable the person to stay in control of their condition, tailored to the person, condition, and severity. Includes how to take medication, recognize symptoms of deterioration and what actions to take” [18 p.78].  “Examples of activities from PRISMS dataset … Written action plan to enable self-adjustment of medications in response to worsening asthma based on symptoms and/or peak flow (Asthma). Specific advice on adjusting insulin dosage, or managing hypoglycaemia (Diabetes)” [18 p.78].  *Update added during data analysis:* *Coding requires explicit mention of the instructions being tailored to the client (unlike ‘information about condition and/or its management’). Said differently, coding requires a clear suggestion that a recorded (written or video) action plan was used (more than just a goal or reminder – a set of step-by-step, how-to instructions), that was tailored to the client (tailored with or by a professional – if ‘with’ a professional [i.e., self-tailored], it must be clear that the professional supported/provided feedback/worked with the client to self-tailor), and was intended to guide the client when they are on their own (not in a professional-guided session).* |
|  | **Regular clinical review** | | “A regular, scheduled review of the person, their condition and self-management, conducted by a healthcare professional” [18 p.78].  “Examples of activities from PRISMS dataset … Regular clinical visits reviewing the person’s condition and self-management (Stroke, Asthma and other long-term conditions)” [18 p.78]. |
|  | **Monitoring of condition with feedback** | | “Monitoring symptoms, behaviours or objective measures related to the long-term condition. Can be done by the person with a long-term condition or by others but the results must be fed back to the patient. Interpretation, decision and/or action is undertaken by the patient, but may be supported by a professional. Professionals may support self-management by reviewing monitored data and providing feedback to the patient” [18 p.78].  “Examples of activities from PRISMS dataset … Daily log completion including peak flow, triggers, and ratings of benefits (Asthma). Patients could send information about self-monitoring drug regimen and physiologic variables to physicians, who reviewed the data and sent personalized recommendations back to the patients (T2 diabetes)” [18 p.78].  *Update added during data analysis: Coding requires explicit mention of clients engaging with data related to self-management/monitoring of their condition.* |
|  | **Practical support with adherence (medication or**  **behavioural)** | | “Provision of practical help to improve a person’s adherence to medication or behaviour change activities” [18 p.78].  “Examples of activities from PRISMS dataset … Diary of medication use and seizures, Dosette medication containers, and prescription refill and appointment-keeping reminders (Epilepsy). Adherence improvement strategies such as taking medication with regularly scheduled activities (Asthma). Weekly reminder telephone calls to perform foot care (T2 diabetes)” [18 p.78].  *Update added during data analysis: Coding requires explicit mention of providing practical support related to adherence beyond what would be expected from ‘regular clinical review’.* |
|  | **Provision of equipment** | | “Provision of equipment to enable, assist or promote self-monitoring and/or self-management of the long-term condition” [18 p.78-79].  “Examples of activities from PRISMS dataset … Bag of supplies to enable foot care (containing soap, towel, socks, mirror, toenail clippers, lotion samples) (T2 diabetes) Provision of a peak flow meter free of charge (Asthma). Home coagulation testing equipment (Stroke)” [18 p.78-79].  *Update added during data analysis: Will not be coded in cases where all clients clearly use their own device and clearly do not receive any new software/features for their device etc. (e.g., will not be coded in the case of sending/receiving SMS text messages through a personally owned device). Sheet-based/physical educational booklets will not count as equipment.* |
|  | **Provision of easy access to advice or support when needed** | | “People with long-term conditions are provided with flexible access to and timely advice from health services in the event of an urgent or non-urgent question or concern arising” [18 p.79].  “Examples of activities from PRISMS dataset … Contact details of specialist nurse helpline for information or support or to advise in the event of clinical deterioration (Stroke). Provision of an out of hours service for advice and support (various long-term conditions)” [18 p.79].  *Update added during data analysis: Coding requires explicit mention of providing clients with access to advice/support beyond what would be expected from ‘regular clinical review’.* |
|  | **Training/**  **rehearsal to communicate with healthcare professionals** | | “Teaching people with long-term conditions to develop communication skills/techniques to improve relationships, better communicate needs, and enhance shared decision making with healthcare professionals. Also supporting/mentoring people with long-term conditions to practice the skills they have been taught” [18 p.79].  “Examples of activities from PRISMS dataset … Strategies for communicating with health-care providers, such as taking a tape recorder to doctors’ visits and recording consultation (Asthma). Community Support Workers from minority ethnic/deprived groups who provide advocacy and support communication with health-care professionals, attending an appointment with the person with the long-term condition (T2 diabetes)” [18 p.79].  *Update added during data analysis: Coding requires explicit mention of providing clients with formal training related to communicating with healthcare professionals (must go beyond the experiential learning which would be expected with ‘regular clinical review’) and/or explicit mention of using a strategy to enhance shared decision making between clients and healthcare professionals.* |
|  | **Training/**  **rehearsal for everyday activities** | | “Teaching people with long-term conditions to develop skills that support everyday activities and/or supporting people with long-term conditions to practice the skills they have been taught” [18 p.79].  “Examples of activities from PRISMS dataset … Occupational therapy activities such as transfers, washing and dressing practice (Stroke). Cognitive rehabilitation (Dementia)” [18 p.79]. |
|  | **Training/**  **rehearsal for practical self-management activities** | | “Teaching people with long-term conditions to develop specific practical skills that will enable them to manage their condition, and/or supporting people with long-term conditions to practice the skills they have been taught” [18 p.79].  “Examples of activities from PRISMS dataset … Inhaler technique instruction (Asthma). Practising foot care procedures (T2 diabetes). Teaching patients to use a home dialysis machine (CKD). Teaching patients to take their own blood pressure (Hypertension)” [18 p.79]. |
|  | **Training/**  **rehearsal for psychological strategies** | | “Teaching people with long-term conditions skills in using psychological strategies to help them better manage the consequences of a long-term condition and/or supporting them to practice the skills they have been taught. May include: problem-solving strategies, relaxation techniques, re-framing, distraction, cognitive restructuring, goal setting and action planning (prompts detailed planning of performance of the behaviour/outcome of the behaviour, NB this does not have to be health behaviour focused)” [18 p.79].  “Examples of activities from PRISMS dataset … Personal goals aimed at reducing risk of further stroke (Stroke). Computerized game which challenges the player to ‘think’ about asthma control. If a problem is noted, the player can create a solution and ‘act’ (Asthma)” [18 p.79]. |
|  | **Social support** | | “Facilitation of social support, where a person feels cared for and supported by others in a social network. May include befriending, peer support, peer mentoring and group socializing” [18 p.79].  “Examples of activities from PRISMS dataset … Encouraging participants to interact and assess their own and their peers’ progress toward managing their diabetes by sharing ideas, advice, and support (T2diabetes). School asthma education to enhance peer understanding/support (Asthma)” [18 p.79]. |
|  | **Lifestyle advice and support** | | “Provision of advice and support around health and lifestyle. Relates to practical advice and support in relation to handling life stressors, NOT psychological elements that relate to handling life stressors (see training/rehearsal in psychological strategies). May include general lifestyle advice and support concerning diet, physical activity, smoking cessation, and alcohol intake” [18 p.79].  “Examples of activities from PRISMS dataset … Assist the parent in smoking cessation (Paediatric asthma). Monthly clinic visits with nutritionist providing advice to enhance physical activity and dietary intake (T2DM). Salt restriction advice (Hypertension)” [18 p.79].  *Update added during data analysis: Coding requires a clear suggestion that both advice and support were provided related to “lifestyle” (the term “lifestyle” must be used explicitly).* |
