# Supplementary material for: Mobile Technology–Based Interventions for Stroke Self-Management Support: Scoping Review
Source: JMIR Mhealth Uhealth. 2023 Dec 6;11:e46558. doi: 10.2196/46558 (PMC10733834; doi:10.2196/46558)
Supplement: Multimedia Appendix 4 [file mhealth_v11i1e46558_app4.docx]

**Multimedia Appendix 4.** Operational definitions used in the directed content analysis related to mobile health functions (objective 3).

| **Code** | **Operational Definition** |
| --- | --- |
| **Educating** | Dictionary definition: “To provide with information” [32].  Examples from past research: “providing information” [30 p.6]; “provide instruction on how to perform behaviour ... provide information on where and when to perform the behaviour … information on consequences of behaviour in general … normative information about others’ behaviour” [31 p.650]; “may inform users by educating them on their disease state or medications they take” [29 p.6]. |
| **Communicating** | Dictionary definition: “To transmit information, thought, or feeling so that it is satisfactorily received or understood” [33].    Examples from past research: “communicating with people that are using this application” [30 p.1070]; “enable communication with a patient’s healthcare team … an app may provide for communication with a patient’s care team through the ability to email a patient’s physician the data from the app. Alternatively, an app may also automatically push data to the physician or automatically integrate it into the patient’s EMR” [29 p.6-7]. |
| **Goal setting** | Dictionary definition: “The process of deciding what you want to achieve or what you want someone else to achieve over a particular period” [34].    Examples from past research: “planning and monitoring the goal of the targets in weight loss … planning and monitoring the goal of the targets in gaining weight … planning and monitoring the goal of the targets in sport or exercise” [30 p.1070]; “mainly consisted of weight loss goals, calorie balance goals, water intake goals, and physical activity goals” [31 p.4]; “goal setting – behaviour … goal setting – outcome … prompt review of behavioural goals … prompt review of outcome goals … set graded tasks … action planning” [31 p.650]. |
| **Monitoring** | Dictionary definition: “To watch, keep track of, or check usually for a special purpose” [35].    Examples from past research: “monitoring weight … monitoring BMI … monitoring calorie usage … monitoring calorie intake … counting steps … sleep monitoring … monitoring heart rate … monitoring health” [30 p.1070]; “allowed the user to track targeted weight-related metrics over time, the majority of which consisted of weight, energy balance, water intake, and quantity of physical activity … could automatically monitor the user’s physical activity without the requirement for manual logging” [31 p.4]; “feedback or monitoring” [30 p.6]; “prompt self-monitoring of behaviour … prompt self-monitoring of behavioural outcomes” [31 p.650]; “Through integrating these same patient-reported outcomes or results of passive data collection mHealth technologies may provide data visualization allowing both patients and physicians can monitor a patient’s condition and track progress over time” [29 p.6]. |
| **Providing feedback** | Dictionary definition: “The transmission of evaluative or corrective information about an action, event, or process to the original or controlling source” [36].    Examples from past research: “provided personalized feedback to the user, such as through virtual meetings with a health coach or through notifications” [31 p.5]; “feedback or monitoring” [30 p.6]; “provide feedback on performance” [31 p.650]. |
| **Reminding** | Dictionary definition: “To put in mind of something: cause to remember” [37].    Examples from past research: “reminding” [30 p.6]; “teach to use prompts/cues” [31 p.650]. |
| **Motivating** | Dictionary definition: “To provide with a motive” [38].    Examples from past research: “motivational strategies including prompts, gamification, or use of rewards (i.e., points for meeting weight goals)” [31 p.4]; “prompting motivation” [30 p.6]; “seek to motivate patients to make better health decisions. This may come in the form of motivational messages that reach patients at the right time, such as when they need to take their medication” [29 p.6]. |
