# Supplementary material for: Mobile Technology–Based Interventions for Stroke Self-Management Support: Scoping Review
Source: JMIR Mhealth Uhealth. 2023 Dec 6;11:e46558. doi: 10.2196/46558 (PMC10733834; doi:10.2196/46558)
Supplement: Multimedia Appendix 5 [file mhealth_v11i1e46558_app5.docx]

**Multimedia Appendix 5.** Intervention characteristics according to selected items from the Template for Intervention Description and Replication checklist. [24]

| **Citation(s)** | **Brief Name** | **Why** | **What (Materials and Procedures)** | **Who Provided** | **How** | **Where** | **When and How Much** | **Tailoring** |
| --- | --- | --- | --- | --- | --- | --- | --- | --- |
| Asano et al [41] | HomeRehab | Goal: To improve  self-reported functional outcomes.    Telerehabilitation rationalized as strategy to improve convenience. | Program type: Physical exercise (self-directed, professional-supported).  Program content: Exercises for upper and lower limb strengthening, seating and standing balance, as well as functional activities.  Hardware/devices: Tablet (iPad), 2 limb sensors, 4 motion sensors, heart rate and blood pressure monitoring set.  Software/features: iPad app (HomeRehab, that allowed clients/caregivers to access demonstration videos, video recording, and real-time biofeedback); videoconference software (Facetime); and data storage software (MySQL).  Other physical materials: Device chargers and tablet stand. | PT/OT | Dyad-based, delivered in-person and  virtually. | Hospital/ home-based in-person component and home-based virtual component. | Before discharge, 1–3 in-person training sessions (1 hour each).  After discharge, 1 in-person set-up session and follow-up (videoconference) sessions as needed.  Delivered over 3  months. | Yes |
| Emmerson et al [42] | Home exercise | Goal: To improve  adherence to home exercise programs and functional  outcomes.    Smart technology  rationalized as strategy to improve access, dosage, and effectiveness. | Program type: Physical exercise (self-directed, professional-supported).  Program content: Exercises for upper limb stretching/range of movement, strengthening, and fine motor coordination that were based on National Stroke Foundation Clinical Guidelines with commentary from OT.  Hardware/devices: Tablet (iPad or similar technology if the client preferred to use their own device).  Software/features: Video recordings of the client performing a collection of exercises; and alarms. | OT | Individual / dyad-based, delivered in-person and  virtually. | Hospital-based in-person component and home-based virtual component. | 1-2 sessions / day.  Delivered over 4 weeks. | Yes |
| Ferrete Ruíz et al [43] | Language recovery apps | Goal: To improve language.  Portable technology rationalized as strategy to improve effectiveness. | Program type: Linguistic exercise (self-directed, professional-supported).  Program content: Strategy for communication and exercises for language rehabilitation.  Hardware/devices: Tablet.  Software/features: 2 apps (Let me Talk®, Appnotize UG for a communicator; Puedo Hablar®, Las Palabras Perdidas for language rehabilitation exercises [it lists the exercises that are well performed and the areas with deficiencies]).  Other physical materials: Diary to record time spent using the apps. | Nurse | Dyad-based, delivered in-person and virtually. | Hospital-based in-person and virtual components. | Delivered over 30 days. | Not Reported |
| Grau-Pellicer et al [44] | Fitlab | Goal: To improve levels of physical activity and walking speed, decrease sedentary time, and enhance health markers.    mHealth technology rationalized as strategy to improve effectiveness. | Program type: Physical exercise (self-directed, professional- and peer-supported).  Program content: Exercises for ambulation, aerobic training, task-oriented training, balance, and stretching.    Hardware/devices: Smartphone and pedometer.  Software/features: 2 apps (Fitlab Training and Fitlab Test [[www.HealthSportlab.com](http://www.healthsportlab.com/)], for supervision of adherence to physical activity using the GPS and accelerometer, completion of questionnaires to assess client mood, effort, recovery, wellness and fatigue, and communication of feedback between client and researcher); and instant messaging software (WhatsApp, for groups of clients to keep in communication, receive feedback, discuss topics related to the exercise program, and motivate each other to keep an active lifestyle). | PT and  researcher | Group-based, delivered in-person and virtually. | Hospital-based in-person component and home-based virtual component. | In-person  sessions 2 / week (1 hour each).  150 minutes / week of moderate physical exercise.  Delivered over 8 weeks. | Not Reported |
| Hankinson et al [45] | GotRhythm | Goal: To improve motor function.  Digital therapeutic technology rationalized as strategy to improve access and effectiveness. | Program type: Physical exercise (professional-directed).  Program content: Music-motor therapy focused on participant-specific movement tasks (both upper and lower limb movements, as well as gross and fine motor skills).  Hardware/devices: Mobile phone (iPhone 5, Apple Inc., CA) and wearable non-intrusive sensors (wireless inertial motion units [IMU], Mbientlab Inc., San Francisco, CA, for wirelessly transmitting acceleration, gyroscope, and magnetometer data).  Software/features: App (GotRhythm, for integrating personal music, wireless wearable sensors, and real-time auditory feedback via a metronome to deliver a tailored Rhythmic Auditory Stimulation protocol and record motor performance throughout training). | Allied health professional | Individual-based, delivered in-person. | Hospital-based in-person component. | 3 sessions (20 minutes each) / week.  Delivered over 6 weeks. | Yes |
| Ifejika et al [46] | Lose It! | Goal: To improve weight reduction, stroke risk factor control, behavioral modification, and program maintenance.    mHealth technology rationalized as strategy to improve access and effectiveness. | Program type: Weight management (self-directed, professional-supported).  Program content: Culturally competent dietary counseling, goal setting, calorie monitoring, and exercise.  Hardware/devices: Smartphone (personally owned).  Software/features: App (Lose It! version 5.2.1, for clients/caregivers to send the research team messages, receive reminder messages via push notifications, search for foods, log calories and exercise, and scan/upload bar codes of grocery items).  Other physical materials: US Department of Health and Human Services Dietary Approaches to Stop Hypertension Eating Plan Guide; American Heart Association cookbooks; American Heart Association reading materials; and measuring cups. | Clinician  and researcher | Individual / dyad-based, delivered in-person and virtually. | Hospital-based in-person component and home-based virtual component. | During month 1, 1 reminder  message / day.  During months 2 and 3, 1 summary of  compliance / week and  reminder messages on missed days.  During months 4 through 6, 1 summary of compliance / week. | Yes |
| Jang & Jang [47] | Finger training app | Goal: To improve  motor function.    Tablet / smartphone apps  rationalized as strategy to improve convenience, access, and effectiveness. | Program type: Physical exercise (self-directed, gamified).  Program content: Exercises for finger training, focused on stretching, flexion, extension, opposition, and thumb abduction.    Hardware/devices: Tablet (Galaxy Note 10.1).  Software/features: App (that included three sections: registration, evaluation, and training [5 training programs, with 1-3 levels each and 20 attempts per level]).  Other physical materials: Checklist that asked clients to track the number of completed training sessions. | OT | Individual-based, delivered in-person and virtually. | Not Reported | 6 sessions (31 minutes each) / week.  Delivered over 4 weeks. | Not Reported |
| Kamal et al [48] | SMS4Stroke | Goal: To improve  medication adherence.    Based on: Health belief model, social cognitive theory, and Coventry, Aberdeen, and London – Refined (CALO-RE) taxonomy of behaviour change techniques.    mHealth technology  rationalized as strategy to improve  intervention effectiveness, access, and cost-effectiveness. | Program type: Medication adherence (self-directed, professional-supported).  Program content: Customized medication reminders and health information messages on the topics of disease, modifiable risk factors, drug information, and importance of optimal drug adherence.    Hardware/devices: Mobile phone (personally owned).  Software/features: Standardized SMS medication reminders customized to the client’s individual prescription; standardized SMS health information messages customized to the client’s medical and drug profile; software to send/receive SMS messages (FrontlineSMS); and phone calling. | Researcher | Individual / dyad-based, delivered in-person and virtually. | Hospital-based in-person component and home-based virtual component. | 1 SMS  reminder for each dose and 2 SMS health information messages / week.  Phone call if no response to SMS medication reminders.  Delivered over 8 weeks. | Yes |
| Kamal et al [49] | Movies4Stroke | Goal: To improve  stroke risk factor control, mortality,  and functional  outcomes.    mHealth technology  rationalized as strategy to improve access. | Program type: Stroke education (self-directed, professional-supported).  Program content: Education focused on an introduction to stroke, emergency response for survival, rehabilitation skills, safe swallowing and speaking skills, medications, and stroke prevention.  Hardware/devices: Tablet and mobile phone (Android, personally owned).  Software/features: App (Movies4Stroke, which included six sets of 5-minute educational videos on various stroke-related topics and pre/post knowledge questions for each set of videos); SMS  messages which reminded the client/caregiver to watch the videos; software to send/receive SMS messages (Frontline); and phone calling (Stroke Helpline).  Other physical materials: Memory chip to transfer the app into the participant’s mobile phone. | Researcher | Dyad-based, delivered in-person and virtually. | Hospital-based in-person component and home-based virtual component. | Videos were presented at 4 time points (enrollment, discharge, first month after discharge, and third month after discharge).  SMS reminder messages 2 / week.  Stroke Helpline active 24/7.  Delivered over 3 months. | Not Reported |
| Kamwesiga et al [50] | F@ce (Face-to-face between the therapist and client, Assessment, Collaboration, and Evaluation) | Goal: To improve  functioning, participation, and  self-efficacy in daily activities.    Telerehabilitation using mobile phones  rationalized as strategy to improve cost-effectiveness and access. | Program type: ADL training (self-directed, professional-supported).  Program content: Education (covering basic knowledge about stroke and rehabilitation), problem-solving (using the Target-Plan-Perform-Prove strategy) and goal setting (using the Canadian Occupational Performance Measure [COPM]).  Hardware/devices: Mobile phone (personally owned).  Software/features: SMS messages that reminded the client/family member to perform the target ADLs; software to send/receive SMS messages; and phone calling.  Other physical materials: Sheet of paper that asked the client to rate their own performance on the target ADLs using a five-point scale. | OT and  researcher | Dyad-based, delivered in-person and virtually. | Home-based in-person and virtual components. | SMS  reminder messages 2 / day and phone calls 2 / week (and more as needed [based on SMS responses]).  Delivered over 8 weeks. | Yes |
| Kang et al [51] | Mirror therapy | Goal: To improve  central facial paresis.    Tablet apps  rationalized as strategy to improve  effectiveness. | Program type: Physical exercise (self-directed, professional-supported).  Program content: Exercises for orofacial mirror therapy.  Hardware/devices: Tablet.  Software/features: App that converted images from right to left and then applied a shade over the half of the screen opposite to the unaffected side (resulting in the client watching the reflection of the unaffected half of the face as if it were the affected half). | SLP | Individual-based, delivered in-person and virtually. | Hospital-based in-person and virtual components. | 2 sessions / day (15 minutes each).  Delivered over 14 days. | Not Reported |
| Kang et al [52] | SHEMA (Stroke Health-Education Mobile App) | Goal: To improve  knowledge of stroke risk factors and health-related quality of life.    Mobile apps  rationalized as strategy to improve access and effectiveness. | Program type: Stroke education (self-directed).  Program content: Education focused on topics related to stroke risk factors: stroke history, heart disease, age, irregular work and sleep patterns, obesity, family history and genetic factors, hyperlipidemia, hypertension, unbalanced diet, diabetes mellitus, changes in ambient temperature, and sex.  Hardware/devices: Smartphone (personally owned).  Software/features: App (SHEMA, that provided clients with stroke-related health information to read). | Researcher | Individual-based, delivered in-person and virtually. | Hospital-based in-person component and home-based virtual component. | 1 session / day (minimum 5 minutes each).  Delivered over 7–14 days. | Yes |
| Kenny et al [53] | Video-guided exercise | Goal: To improve motor outcomes  and self-efficacy.    Tablet technology rationalized as strategy to improve access  and effectiveness. | Program type: Physical exercise (self-directed, professional-supported).  Program content: Exercises for upper limb training.    Hardware/devices: Tablet.  Software/features: Video recordings (both of the therapist performing the exercises as well as of the client performing the exercises with verbal prompts from the therapist).  Other physical materials: User guide, diary for recording time spent exercising, charger, and tablet stand. | PT | Individual-based, delivered in-person and virtually. | Hospital-based in-person and virtual components. | Videos were watched 3 times  before  practicing.  Practiced exercises as often as they wished.  Delivered over 4 weeks (or until discharge if less than 4 weeks). | Yes |
| Kim et al [54] | ZyMi | Goal: To improve  balance and gait abilities.    Smartphone apps  rationalized as strategy to improve  convenience. | Program type: Physical exercise (professional-directed).  Program content: Exercises for rhythmic auditory stimulation gait training focused on practicing forward walking, backward walking, side walking, standing up and sitting down from a chair, crossing obstacles, and climbing upstairs and downstairs to the metronome beat.  Hardware/devices: Smartphone (Android).  Software/features: App (ZyMi Metronome FREE).  Other physical materials: Earphones. | Researcher | Individual-based, delivered in-person. | Hospital-based in-person component. | 3 sessions / week (30 minutes each).  Delivered over 5 weeks. | Yes |
| Labovitz et al [55] | AiCure | Goal: To improve  medication adherence.    Artificial intelligence via smartphones  rationalized as strategy to improve  intervention effectiveness, access, and cost-effectiveness. | Program type: Medication adherence (self-directed, professional-supported).  Program content: Focused on verifying drug administration.  Hardware/devices: Smartphone.  Software/features: Artificial intelligence app (AiCure, that provided medication reminders, dosing instructions, visual confirmation of medication ingestion [via facial recognition], real-time data encryption and transmission, and connection to clinic staff via SMS/email). | Clinic staff | Individual-based, delivered in-person and virtually. | Hospital-based in-person component and home-based virtual component. | Clinic visits occurred at 4 time points (baseline and weeks 4, 8, and 12).  Clinic staff received SMS messages/ emails if doses were missed, late, or based on incorrect usage. | Not Reported |
| Lakshminarayan et al [56] | Hypertension self-management | Goal: To improve  hypertension control and medication adherence.    mHealth  technology  rationalized as strategy to improve  effectiveness. | Program type: Risk factor management (self-directed, professional-supported).  Program content: Education, blood pressure self-monitoring, and communication with professionals regarding medication and blood pressure.    Hardware/devices: Smartphone (iPhone) and upper arm Withings Wireless Blood Pressure Monitor (Nokia).  Software/features: App that allowed the wireless monitor to interface with the smartphone and transmit data to a database; phone calling; and email. | Nurse,  physician, and pharmacist | Individual-based, delivered in-person and virtually. | Hospital-based in-person component and home-based virtual component. | Blood pressure monitoring session 1 / day and phone calls or emails as needed (based on  monitored data).  Delivered over 90 days (or up to 6 months). | Not Reported |
| Maresca et al [57] | VRR (Virtual Reality Rehabilitation System)-Tablet | Goal: To improve linguistic function, psychological well-being, quality of life, and mood.    Telerehabilitation rationalized as strategy to improve intervention effectiveness, access, and cost-effectiveness. | Program type: Linguistic exercise (self-directed, professional-supported, gamified).  Program content: Exercises for naming, composing, writing, and rewriting words.    Hardware/devices: Tablet (VRRS-Tablet).  Software/features: Software that offers progressively difficult linguistic exercises; and software for videoconferencing, data transmission, and remote control. | Neuropsychologist | Individual / dyad-based, delivered in-person and virtually. | Hospital-based in-person component and home-based virtual component. | 5 exercise  sessions  / week (50 minutes  each).  Delivered over 6 months.  Phase 1 (first 12 weeks) took place at the hospital and Phase 2 (second 12 weeks) took  place at  home.  During Phase 2, 2 videoconference sessions / week. | Yes |
| Moon et al [58] | Orofacial muscle exercise program | Goal: To improve  swallowing function and intervention satisfaction.    Smartphone  technology  rationalized as strategy to improve  convenience,  satisfaction, and effectiveness. | Program type: Physical exercise (self-directed, professional-supported).  Program content: Exercises for orofacial training (e.g., opening the mouth wide, moving the jaw from side to side with the mouth wide open, moving the jaw backward and forward with the mouth wide open, smiling).  Hardware/devices: Smartphone.  Software/features: 7-minute video recording of the therapist performing the exercises. | OT | Individual-based, delivered in-person and virtually. | Hospital-based in-person and virtual components. | 12 sessions (20 minutes each) over 4 weeks.  During each session, the  video was watched 3 times while practicing. | Not Reported |
| Øra et al [59] | Augmented  speech and language therapy | Goal: To improve expressive language function.    Telerehabilitation  rationalized as  strategy to  improve access,  dosage, and convenience. | Program type: Linguistic exercise (professional-directed).  Program content: Exercises for spoken language, focusing on word production, picture naming, and discussion about familiar topics, based on the Newcastle University Aphasia Therapy Resources (NUMA) and Sareptas afasikrukke and Lexia (SLP computer-based training program).  Hardware/devices: Laptop.  Software/features: Software for remote control (LogMeIn) and videoconferencing (CiscoJabber/Acano).  Other physical materials: External speakers; wide-angle web camera; and text/pictures from the internet. | SLP | Individual / group-based, delivered in-person and virtually. | Hospital-based in-person component and home-based virtual component. | 5 sessions / week.  Delivered over 4 weeks. | Yes |
| Pandian et al [60] | SPRINT INDIA (Secondary Prevention by Structured Semi-Interactive Stroke Prevention Package in India) | Goal: To improve secondary stroke prevention.  mHealth technology rationalized as strategy to improve effectiveness. | Program type: Risk factor management (self-directed, professional-supported).  Program content: Education (related to stroke in general, risk factors, atrial fibrillation, medication adherence, blood pressure and glucose control, physical activity, nutrition, and rehabilitation), workbook activities, and follow-up with a research coordinator.  Hardware/devices: Mobile phone (personally owned).  Software/features: 68 SMS text messages, 6 (2–4 minute long) video messages (translated into 11 Indian languages and developed with a positive sentiment and conversational tone), and phone calling.  Other physical materials: Printed workbook (12 chapters, 8 stories of stroke survivors, and 15 interactive activities, such as board games, pair matching, true-or-false questions with answers, simple physical exercises, and an exercise calendar). | Researcher | Individual / dyad-based, delivered in-person and virtually. | Hospital-based in-person component and home-based virtual component. | After enrollment, education session in-person.  SMS messages 1 / day for 6 weeks, then 2 / week until 6 months, then 1 / week until 1 year.  Video messages 1 / week for 6 weeks, then 1 / month until 1 year.  Workbook activities completed within 6 weeks then revised 1 / month.  Phone call if no response to SMS/video messages. | Yes |
| Radomski [40] | WARMS (Web-based Adherence Reinforcement Monitoring System) | Goal: To improve adherence to  treatment  recommendations, independence, caregiver burden,  and automaticity  of self-care tasks.    Based on: Proposed ecological model of adherence to rehabilitation  treatment  recommendations  and self-determination theory.    Smartphone technology rationalized as strategy to improve  effectiveness. | Program type: ADL training (self-directed, professional-supported).  Program content: Self-care habit training focused on developing and adhering to a morning self-care checklist (performing a list of ADLs such as tooth brushing, dressing, eating breakfast etc.).    Hardware/devices: Smartphone (Cingular 8125 Pocket PC).  Software/features: WARMS system, which consists of a collection of questionnaires (that encourage adherence to the self-care checklist, provide reinforcing messages, assess the level of assistance and concentration required to perform tasks, and offer reminders to recharge the smartphone); Clinical Connection Software; and Skaffold.com (web-based software platform that holds, schedules, sends, and receives responses to questionnaire data).  Other physical materials: Morning self-care checklist sheet and placemat (two-sided, laminated sheet with pictorial and narrative WARMS instructions). | OT/ researcher | Dyad-based, delivered in-person and virtually. | Home-based in-person and virtual components. | Home visits occurred at 2 time points (within a week of discharge and at the end of the intervention).  After discharge, questions and prompts were sent daily, over 4-5 weeks. | Yes |
| Sarfo et al [61]; Sarfo et al [62] | PINGS (Phone-based Intervention under Nurse Guidance after Stroke) | Goal: To improve blood pressure control, medication adherence, hypertension management competence, autonomous self-regulation, and knowledge of hypertension and stroke.    Based on: Self-determination theory.    mHealth technology rationalized as strategy to improve access. | Program type: Risk factor management (self-directed, professional-supported).  Program content: Lifestyle education, blood pressure self-monitoring, and reporting to a nurse.    Hardware/devices: Smartphone and Blue-toothed UA-767 Plus BT blood pressure device.  Software/features: App for monitoring and reporting blood pressure measurements and medication intake; motivational and reinforcement SMS messages (reminders related to blood pressure monitoring, medication adherence, and lifestyle tips); and a secure web page (hosted at the Medical University of South Carolina) that calculated the mean blood pressure and daily medication intake adherence scores then forwarded summary reports. | Nurse | Individual-based, delivered in-person and virtually. | Hospital-based in-person component and home-based virtual component. | Blood pressure monitoring sessions 2 / day, SMS messages between 1 / day to several times / week (based on adherence data).  Delivered over 3 months. | Yes |
| Tomori et al [63] | ADOC (Aid for Decision-making in Occupation Choice) | Goal: To improve  health-related quality of life, motor recovery, independence,  and patient satisfaction, as well as reduce duration of stay.    Based on: International Classification of Functioning, Disability, and Health (ICF) framework. | Program type: ADL training (professional-directed).  Program content: Occupation-based goal setting focused on shared decision-making and occupation-based practice (e.g., using chopsticks to eat food, cooking, knitting).    Hardware/devices: Tablet (iPad).  Software/features: App (ADOC, that consists of 94 illustrations describing different ADLs of which the client could choose from). | OT | Individual-based, delivered in-person. | Hospital-based in-person component. | During the hospital stay, more than two-thirds of the intervention time was allocated to real occupation-based  practice and as  appropriate, the remaining one-third was allocated to basic  functional  exercises and simulated occupation-based  practice. | Yes |
| Vahlberg et al [64]; Vahlberg et al [65] | STROKEWALK | Goal: To improve overall mobility,  including walking endurance and lower body strength, as well as body composition, cardiometabolic risk markers, and self-reported health.    mHealth technology rationalized as strategy to improve intervention effectiveness, convenience, and cost-effectiveness. | Program type: Physical exercise (self-directed, professional-supported).  Program content: Exercises for outdoor community walking and strength training.  Hardware/devices: Mobile phone and pedometer/moving sensor (YamaxLS20000).    Software/features: SMS messages that provided instruction on what and how to exercise to increase walking endurance and improve lower body strength (instructions gradually increased in intensity and frequency); and an internet service ([www.intime.nu](http://www.intime.nu/)) that delivered the SMS messages.  Other physical materials: Training diary. | PT | Individual-based, delivered virtually. | Home-based virtual component. | 1 SMS message / day and 6 exercise sessions / week.  Delivered over 3 months.  Pedometer  was worn at all times  (except for when sleeping, bathing, or swimming). | Yes |
| Wan et al [66]; Wang et al [67] | CRS-HBM (Comprehensive Reminder System based on the Health Belief Model) | Goal: To prevent recurrent stroke by improving  health behaviors and blood pressure control.    Based on: Health belief model. | Program type: Risk factor management (self-directed, professional-supported).  Program content: Health belief education, goal setting, blood pressure self-monitoring, and self-evaluation.  Hardware/devices: Mobile phone (personally owned).  Software/features: SMS reminders (regarding outpatient clinic visits, lifestyle modification [e.g., physical activities, low-salt diet, medication adherence, and blood pressure checkup], the establishment of health belief, and possible climate changes and precautions [e.g., keep warm and prevent falls]); website to import client data and send automated SMS reminders; and phone calling.  Other physical materials: Educational handbook (based on the health belief model) containing goal setting calendar memos for self-management, blood pressure self-monitoring sheet, and self-evaluation chart. | Nurse | Individual-based, delivered in-person and virtually. | Hospital-based in-person component and home-based virtual component. | Education sessions  occurred at 5 time points  (1 in-person  session [2 days before discharge; 20-30 minutes] and 4 virtual  sessions [via phone call; 1 week and 1,3, and 6 months after discharge; minimum 15 minutes each]).  After discharge, 1 SMS reminder / week, over 6 months. | Yes |
| Wang et al [68] | Secondary stroke prevention | Goal: To prevent recurrent stroke by improving  blood pressure control and health self-management ability.  mHealth technology rationalized as strategy to improve effectiveness. | Program type: Risk factor management (self-directed, professional-supported).  Program content: Health education (related to stroke prevention and high-risk factors), monitoring health data (blood pressure, blood glucose, sleep, step count, mileage, calories, and exercise), medication adherence, and early warning management.  Hardware/devices: Smartphone (personally owned), Bluetooth sphygmomanometer (Yuwell 680A), Bluetooth blood glucose meter (LipidEx3B), and wearable bracelet.  Software/features: App (Ekangwang, that connected to the monitoring devices, stored information [such as their exercise plan and their physician’s advice in the hospital], and enabled remote guidance [sent push notifications]); platforms for data management (data was logged in the Regional Health Monitoring Network Platform Based on Mobile End Devices at their hospital and their National Stroke Data Center); and phone calling. | Brain and heart health manager (BHHM) and physician | Individual-based, delivered in-person and virtually. | Hospital-based in-person component and home-based virtual component. | Before discharge, 1 in-person session.  After discharge, push notifications 3-7 / week and phone calls as needed (based on monitored health data).  Delivered over 6 months. | Yes |

^a^Abbreviations: ADL, activity of daily living; app, application; GPS, global positioning system; mHealth, mobile health; OT, occupational therapist; PC, personal computer; PT, physical therapist; SLP, speech-language pathologist; SMS, short message service.
