# Supplementary figures and images for: Mobile Technology–Based Interventions for Stroke Self-Management Support: Scoping Review
Source: JMIR Mhealth Uhealth. 2023 Dec 6;11:e46558. doi: 10.2196/46558 (PMC10733834; doi:10.2196/46558)

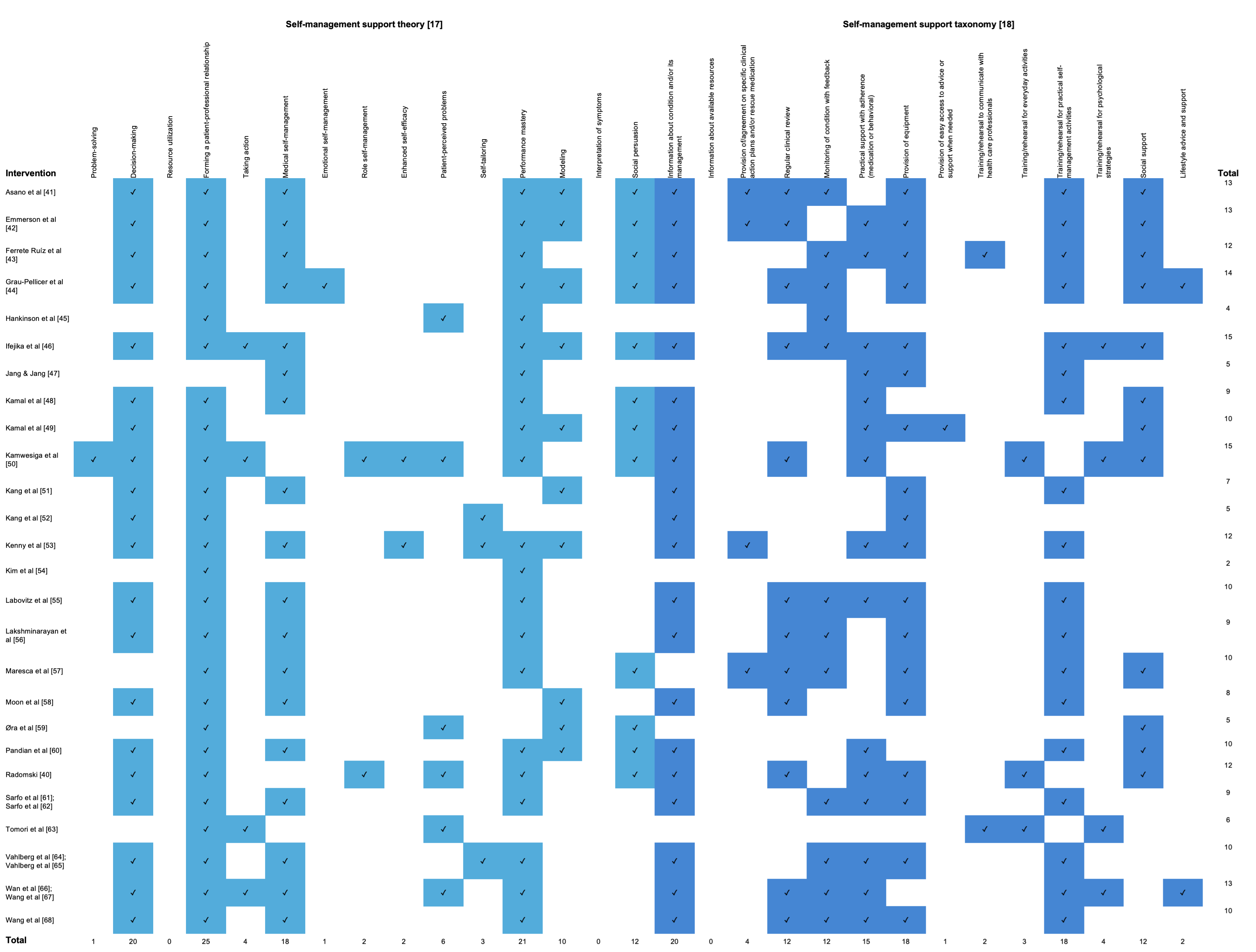

Supplement: Multimedia Appendix 6 [file mhealth_v11i1e46558_app6.png]
